# Highly Transparent and Polarization-Maintained Terahertz Plasmonic Metamaterials Based on Metal-Wire-Woven Hole Arrays: Fundamentals and Characterization of Transmission Spectral Peaks

**DOI:** 10.3390/ma15051871

**Published:** 2022-03-02

**Authors:** Borwen You, Ja-Yu Lu, Po-Lun Chen, Tun-Yao Hung, Chin-Ping Yu

**Affiliations:** 1 Department of Physics, National Changhua University of Education, No. 1 Jinde Road, Changhua City 50007, Taiwan; 2 Department of Applied Physics, Faculty of Pure and Applied Sciences, University of Tsukuba, Tennodai 1-1-1, Tsukuba 305-8573, Ibaraki, Japan; 3 Department of Photonics, National Cheng Kung University, No. 1 University Road, Tainan 70101, Taiwan; r09941156@ntu.edu.tw; 4 Department of Photonics, National Sun Yat-Sen University, Kaohsiung 80424, Taiwan; tunyaohung.ee09@nycu.edu.tw (T.-Y.H.); cpyu@faculty.nsysu.edu.tw (C.-P.Y.)

**Keywords:** artificial material, waveguide mode, plasmonic metamaterial, periodic metal structure, terahertz radiation, submillimeter wave

## Abstract

Metal-hole-supported terahertz (THz) waves through the structure of a metal-wire-woven hole array (MWW-HA) present high-frequency-passed transmittance spectra of one plasmonic metamaterial with artificial plasmonic frequencies, which are inversely proportional to metal-hole widths. For the transmitted THz waves of MWW-HA, transverse-electric (TE) and transverse-magnetic (TM) waveguide modes mix within a symmetric metal-hole boundary. THz resonance waves transversely crossing the holes of MWW-HA are experimentally characterized with spectral peaks in the frequency range of 0.1–2 THz that are correlated with aperture sizes, unit-cell-hole widths, metal-wire thicknesses, and wire-bending angles. The metal-hole-transported resonance waves of MWW-HA are dominated by TE waveguide modes instead of TM ones because a hole width of MWW-HA is approximate to the half wavelength of a resonance wave. The round metal edges of the woven metal wires can minimize the effective optical length of a thick metal hole to transmit THz resonance waves, thereby resulting the smallest rotation angle of linear polarization and high transmittance up to 0.94. An MWW-HA structure is therefore reliable for supporting metal-hole resonance waves with low resistance, whereas a metal-slab-perforated hole array cannot achieve the same result.

## 1. Introduction

Metal thin films compositing two-dimensional (2D) hole arrays with subwavelength-scaled aperture widths and nano-micrometers of thicknesses facilitate the study of surface plasmon polaritons (SPPs) in the terahertz (THz) frequency range [1]. Those THz waves supported by a metal-hole-array (MHA) structure are also called surface resonance waves (SRWs) because of the transmission-resonance effect of metal surface waves [2,3]. Their spectroscopic feature [4] entails a distinctly extraordinary transmission (EOT), as explained by the Fano model [5] or indicated as the Fano interference effect [3]. The EOT of THz SRW has sharp peaks in transmission spectra with much higher transmittance than that of metal-hole guided THz waves, i.e., metal-hole waveguide modes [3,4]. For the EOT of THz SRW, high local-field intensity accumulates inside MHA structures, consequently forming one artificial material of THz plasmonics, i.e., THz plasmonic metamaterials.

The local-field accumulation for the EOT of THz SRW eventually reradiates along the optic axial line and transmits through the MHA-based THz plasmonic metamaterials, which are different from those SRWs or SPPs generated at visible-infrared ray (VIS-IR) bands. The local-field accumulation of VIS-IR SPP generation, also called the surface-plasmonic-resonance (SPR) effect, are strongly trapped at dielectric-metal interfaces without obvious radiation along the optic axial line or at a far field range. The VIS-IR SPR effect thus has the potential to realize fast photo-switches [6] and photo-enhancers for solar cells [7].

The THz SRW intensity on an MHA layer can be further controlled by the concepts of super-unit cells [8,9] and by fine tuning the hole geometry, such as through symmetry [10], interspace [11], and metal thickness [12]. Such a controlling concept is straightforward for changing the dimensions of unit-cell structures [13] and performs spectral modification [14]. However, the manipulation of THz SRWs for photonic applications (such as waveguides, near-field confinement, and optical sensing reactors) is still constrained because a large area of a deformable and bendable MHA is required for a flexible operation. For example, THz plasmonic metamaterials, composed of one-dimensional periodic metal strips on a polymer bulk, have been used as THz fiber cladding for a bendable waveguide feature [15]. 

Deforming an MHA entails sufficient metal thickness for robustness at a wide bending range. By contrast, the metal thicknesses of MHAs to ascertain the EOT of THz SRW should be sufficiently smaller than the wavelengths for approximating the metal skin depths of nanometric scales [1,3]. Moreover, the EOT of THz SRW cannot flexibly exist because the exact metal-hole pitch, amount, and orientation angle of wave excitation are demanded as the structural or optical criteria [4,16]. As the EOT of THz SRW fails to be excited from MHAs, only the metal-hole waveguide modes are found in high-frequency-passed transmittance spectra, whose intensity is relatively weaker than the EOT of THz SRW [3]. Therefore, bendable and robust MHAs with a high intensity of metal-hole-supported THz waves are required for THz photonic applications. 

A metal-wire-woven hole array (MWW-HA) is a composite with metal wires based on weaving technology. The MWW-HA structure is robustly bendable and deformable for a large area and volume. It is thus considered as one plasmonic metamaterial with an MHA structure in the THz frequency region, and its spectroscopic features can be studied. The spectral dips of the MWW-HAs in the THz frequency range have been observed and are called Wood’s anomalies [17]. However, the spectral dip field of MWW-HA stops at the bent metal wires without being supported within the metal holes [18]. Although the abnormal group velocity of the THz wave is performed by the metal holes of an MWW-HA structure [17], the geometric factors of an MWW-HA unit have not yet been expressed for the metal-hole wave guidance. 

In this presentation, the unit structure of MWW-HA is specifically modeled with an aperture size, a unit-cell-hole width, a metal-wire thickness, and a wire-bending angle. Metal-hole-supported THz waves through MWW-HAs and based on these four modeling parameters are characterized at the transmission spectral peaks, which are explained, in theory, by metal-hole waveguide modes with a transverse resonance field. The metal-slab-perforated MHAs are used as standard THz plasmonic metamaterials to calibrate the THz spectroscopic measurement to derive the metal-hole waveguide principle of MWW-HA, in theory and in experiments. Three spectral features of the MWW-HA resonance waves are discovered in experiments, including the highest transmittance near the rejection band, the spectral peak splitting effect of resonance waves, and highly polarization-maintained transmission. Such characteristics of THz resonance waves through an MWW-HA structure are statistically analyzed by various structural parameters, and by the measured results of power transmittance and waveform phases. The relevant contents, organized with three sections in this presentation, involve Materials and Methods, Results, and Discussion. The metal-hole configuration, the principle of metal-hole-guided THz waves, and the measurement of an MWW-HA structure are presented in the section Materials and Methods. The spectral features and transmittance performance of metal-hole-supported THz resonance waves are then revealed in the Result section. In the Discussion section, metal-hole supported THz resonance waves on an MWW-HA structure are further analyzed for the spectral-peak splitting effect, the interaction response of woven metal wires, and power flow distributions, which soundly clarify their fundamentals and characterizations. 

## 2. Materials and Methods

### 2.1. Configuration of a Metal-Wire-Woven Hole Array

**Figure 1:**
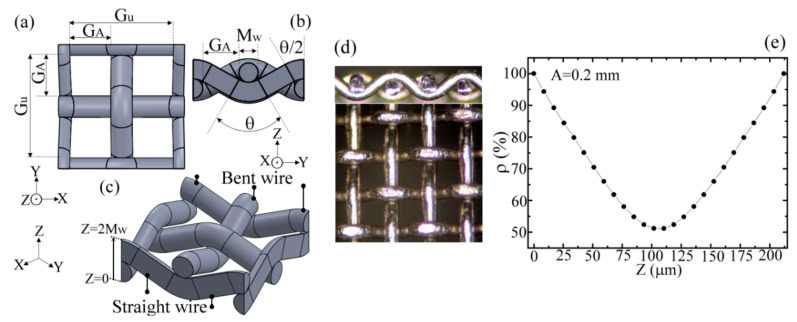
(**a**) top, (**b**) side, (**c**) 3D views and (**d**) microscopic photographs of MWW-HA structure; (**e**) *Z*-axial dependent planar porosity of MWW-HA structure.

The mechanical modeling of an MWW-HA unit cell is illustrated in Figure 1a–c for the top, side, and 3D views, respectively, which correlate to the microscopic photographs of MWW-HA in Figure 1d. The unit cell has four square holes with the configuration of a 2 × 2 aperture array, where an aperture width is denoted as *G_A_* and is consistent at the *X* and *Y* axes, as shown in Figure 1a. In addition, a unit-cell-hole width, denoted by *G_u_*, covers the sizes of two adjacent apertures and one metal-wire width (*M_w_*), i.e., *G_u_* = 2*G_A_* + *M_w_*, as shown in Figure 1a. The *G_u_* value is substantial for THz waves transmitting through the structural thickness of 2*M_w_*, as shown in Figure 1a,b, because two pairs of crossed metal wires, constructing one aperture, *G_A_*, are not wholly at the same *X–Y* plane, i.e., they are at different *Z*-axial locations.

For an MWW-HA, all the structural metal wires bend to support periodic holes, and each metal wire, called a metal woven wire, is composed of the straight- and bent-wire sections in the mechanical modeling, as shown in Figure 1b,c. The mechanical modeling derives the parameters of a curvature radius (*R_C_*) and a bending angle (*θ*) for a bending section of the metal wire to optimize the wire-woven configuration based on the *M_w_* and *G_A_* parameters, as shown in Figure 1b. In the study, five MWW-HAs, constructed by stainless-steel wires, were used, as listed in Table 1. 

When a THz wave transmits through the unit cell of an MWW-HA, the *Z*-axial-dependent planar porosity, *ρ*(*z*), is not constant as a metal-slab-perforated MHA. Figure 1e illustrates the *ρ*(*z*) curve of the 0.20-mm *G_A_* MWW-HA as an example, and each *ρ* value at a *Z* axial location is evaluated within a sliced thickness of 8 μm and a side width of 0.612 mm (2*G_A_* + 2*M_w_*). The volume porosity *ρ_v_* (71%, Table 1) is different from the planar porosity of *ρ*(*z*) in Figure 1e. A lowest *ρ* value passes the THz waves at the MWW-HA structural plane, *z* = *M_w_*. Thus, the metal-hole-guided THz waves through an MWW-HA structure have more complex variations of amplitude and phase than those of metal-slab-perforated MHAs. 

### 2.2. Principle of Metal-Hole-Guided THz Waves

**Figure 2:**
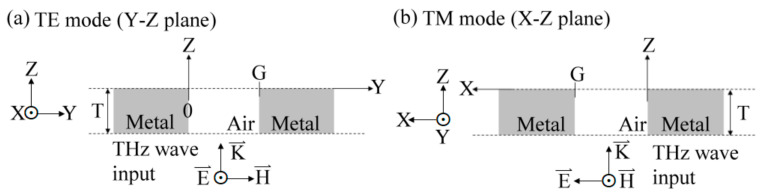
Boundaries, electric, and magnetic field configurations of metal-hole waveguide modes: (**a**) TE and (**b**) TM modes.

Symmetric holes that are perforated on metal slabs with circular or square shapes are the standard conformations to implement the metal-hole-guided THz waves. Stable electric fields inside the holes, called the metal-hole waveguide modes, can be written as [19]:

(1) $$\vec{E}\left(x,y,z\right)=\sum_{m}A_{m}\left(z\right)\cdot E_{m}\left(x,y\right)e^{i\left(2\pi \nu t-\beta_{m}z\right)},$$

where *m*, $\beta_{m}$, ν, and $E_{m}\left(x,y\right)$ are the waveguide mode number, propagation constant, THz frequency, and the amplitude wavefunction of the *m*th mode, respectively. The axis of THz wave propagation is along the *Z* axis, which is perpendicular to the *X–Y* plane. The summation of amplitude constant, $A_{m}$, denotes the wave superposition of all the waveguide modes, including *TE* and *TM* modes. The coupling coefficient from free space to metal-hole waveguide mode is a *z*-dependent function and affects the overall amplitude, $\left|\vec{E}\right|=\sum_{m}A_{m}\left(z\right)\cdot E_{m}\left(x,y\right)$, as shown in Equation (1). On the *X–Y* transverse plane of wave guidance, the amplitude of the metal-hole waveguide mode should be defined with the amplitude product of *TE* and *TM* modes due to the symmetry of the metal hole. The amplitude of a metal-hole waveguide mode is written as:

(2) $$\left|\vec{E}\right|=\int_{0}^{T}\left|{\vec{E}}_{TE}\right|\cdot \left|{\vec{E}}_{TM}\right|dz,$$

where *T*, ${\vec{E}}_{TE}$, and ${\vec{E}}_{TM}$ denote the metal thickness of a structural aperture, electric waves of *TE*, and *TM* waveguide modes, respectively. Figure 2 illustrates the configurations of electric ($\vec{E}$) and magnetic ($\vec{H}$) field vectors for illuminating a transverse electromagnetic wave at one metal hole to perform the *TE* and *TM* waveguide modes, where $\vec{E}$ and $\vec{H}$ are along the *X* and *Y* axes, respectively. The electric field amplitude of the guided TE waves can be written as:

(3) $${\vec{E}}_{TE}=E_{x}\left(y,\;z,\;t\right)=\sin\left(hy\right)\cdot \exp\left[i\left(2\pi \nu t-\beta z\right)\right],$$

where $\sin\left(hy\right)$ is the amplitude wave function for 0 < *y* < *G* because the THz field does not exist inside the metal and satisfies the continuous field at the air–metal interface. Based on the wave equation of a layered dielectric structure that consists of homogeneous and isotropic materials, h is related to the propagation constants by:

(4) $$h=\sqrt{K_{0}^{2}-\beta^{2}},\;i.e.,\;\beta^{2}=K_{0}^{2}-h^{2}.$$

At the metal–air interface of *Y* = *G* in Figure 2a, the electric wave amplitude of a TE mode is zero, $\sin\left(hG\right)=0$. The propagation constant of a TE mode, *h*, is obtained as:

(5) h=mπG, m=1, 2, 3…

For TE waves passing through the metal hole, the criterion of a propagation constant is β>0. Based on Equations (4) and (5), the wavelength (λ) of a TE waveguide mode satisfies the condition:

(6) G>λ/2

When an electric field resonance occurs within the metal hole, the propagation constant approximates zero, β~0. The resonance wavelength ($\lambda_{c}$) has the approximation relation with a *G* magnitude:

(7) $$G≅\lambda_{c}/2.$$

Equations (6) and (7) indicate that the half wavelength value of a metal-hole guided TE mode should be sufficiently smaller than the metal hole width (G). 

The magnetic-field amplitude of a metal-hole-guided TM wave for 0 < *x* < *G*, as shown in Figure 2b, is defined as:

(8) $$H_{y}\left(x,\;z,\;t\right)=\sin\left(hx\right)\cdot \exp\left[i\left(2\pi \nu t-\beta z\right)\right].$$

The corresponding electric field amplitudes at the *X* and *Z* axes are shown in Equations (9) and (10), respectively.

(9) $$E_{x}\left(x\right)=\frac{i}{2\pi \nu \mu }\cdot \frac{\partial }{\partial z}H_{y}\left(x,\;z,\;t\right)$$

(10) $$E_{z}\left(x\right)=-\frac{i}{2\pi \nu \mu }\cdot \frac{\partial }{\partial x}H_{y}\left(x,\;z,\;t\right)$$

Based on Equations (8)–(10), the electric fields of TM polarized waves are presented as:

(11) $$E_{x}\left(x,\;z,\;t\right)=\frac{1}{2\pi \nu \mu }\cdot \sin\left(\frac{m\pi }{G}x\right)\cdot \beta \cdot e^{i\left(2\pi \nu t-\beta z\right)}$$

(12) Ezx, z, t=m2νμG·cosmπGxei2πνt−βz+3π2

For TM resonance waves (β~0), Equation (11) shows that the transverse electric field in the *X* axis equals zero, $E_{x}\left(x,\;z,\;t\right)=0$. Based on Equation (12) and β~0, a *Z*-axial electric field distribution of TM resonance waves exists at the metal hole surface, *x* = 0 and *x* = *G*, as shown in Equation (13).

(13) Ezx, z, t=m2νμG·cosmπGxei2πνt+3π2

The TM modes guided through a metal hole are eventually not constrained by the metal hole width, *G*, which is different from that of the TE modes, as shown in Equations (6) and (7).

### 2.3. Measurement of Metal-Hole-Guided THz Waves

**Figure 3:**
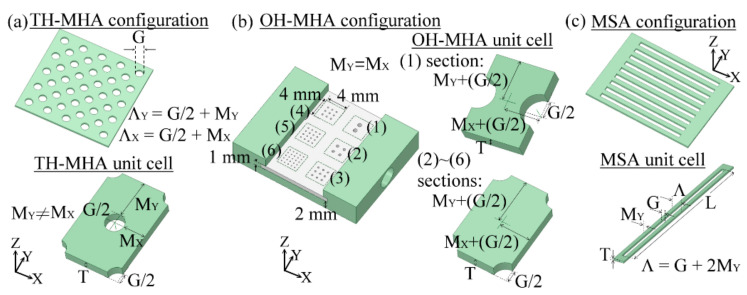
Mechanical drawing of (**a**) TH-MHA, (**b**) OH-MHA, and (**c**) MSA structures.

To approve metal-hole guided THz waves with the electric field properties in Equations (6), (7) and (13), two types of MHAs, as shown in Figure 3a,b, and one metal-slit-array (MSA), as shown in Figure 3c, were prepared. The two types of MHAs include one- and two-hole unit cell structures denoted by OH-MHA and TH-MHA, respectively. The waveguide performance of the three metal-hole structures (Figure 3) is standard to calibrate that of an MWW-HA structure in the experiments. Table 2 lists all the geometric dimensions, including a circular hole or a slit width (*G*), 2D-hole interspaces (*M_X_, M_Y_*), 1D slit interspace (*M_Y_*), metal thickness (*T*), and structural porosity (*ρ_v_*). 

The metal-hole configuration and unit-cell structure of a TH-MHA are presented in Figure 3a. The metal-hole interspace of a TH-MHA at the *X*- and *Y*-axes are different, i.e., *M_X_* ≠ *M_Y_*. For OH-MHAs, the *X*- and *Y*-axial metal-hole interspaces are consistent, as shown in Figure 3b, i.e., *M_X_ = M_Y_*. The parameters of *M_X_* and *M_Y_* can control the structural porosities, *ρ_v_*. The *ρ_v_* values of OH-MHAs were designed from the specified *M_X,Y_* values in Table 2 to be relatively lower than those of TH-MHAs. Comparing the transmittance performance of metal-hole waveguides shows that the information of *ρ_v_* is essential for the analysis. 

Based on Equations (6) and (7), the *G* ranges of OH- and TH-MHAs should be sufficiently large to observe the frequency or wavelength variation of THz resonance waves through various metal holes. Five *G* values of the TH-MHA structure, 0.18–0.75 mm, were obtained from five stainless-steel plates with the *T* range of 0.37–0.81 mm. For OH-MHAs, six *G* values (0.18–0.75 mm) were prepared on the same aluminum metal plate, but at six different sections, which are denoted as Sections (1)–(6) in Table 2 and Figure 3b. The six OH-MHAs have the same *T* value but different *G* and *M_X,Y_* values. Sections (1)–(6) are defined by square areas with side lengths of 4 mm, which can be fully covered by a 4 mm wide THz-wave beam size. The 4 × 4 mm^2^ square area is also used to estimate *ρ_v_* values of OH-MHAs. Owing to the approximate *ρ_v_* values, 4–5.5%, among the six OH-MHAs in the fixed square area, the 0.75-mm *G* OH-MHA in Section (1) only has two metal holes to interact with the THz waves, and the corresponding unit-cell structure of OH-MHA is different from that in Sections (2)–(6), as shown in Figure 3b. Metal-hole waveguide modes are workable through the metal-hole structures of Sections (1)–(6) without any limitation, such as the metal-hole amount, arrangement, and THz wave beam size [4].

Two OH-MHAs with different thicknesses were fabricated from two individual aluminum metal plates that were assembled as in the configuration of Figure 3b. One thickness, *T*, is 2 mm, and the other is 1 mm. The two aluminum plates were fixed and perforated together to obtain the consistent arrangement and sizes of metal holes for the 1- and 2-mm *T* OH-MHAs. Thus, the thickness-dependent waveguiding performances of a metal-hole can be observed on the OH-MHA structure.

To express the amplitude of metal-hole waveguide modes in Equations (1) and (2), the MSA structure was used to mimic the 1D boundary of a circular-hole diameter at the *X* or *Y* axis. The configuration and unit-cell structure of MSA are presented in Figure 3c. Table 2 shows that the structural parameters of an MSA unit are *G*, *M_Y_*, *T*, and *ρ_v_*, which were perforated from four thicknesses of brass plates. In measuring power transmittance, one MSA was rotated with the *Z* axis to make input THz wave polarization at *X* axis parallel and perpendicular to the slit axis, respectively, performing the spectra of TE and TM waveguide modes on each MSA.

**Figure 4:**
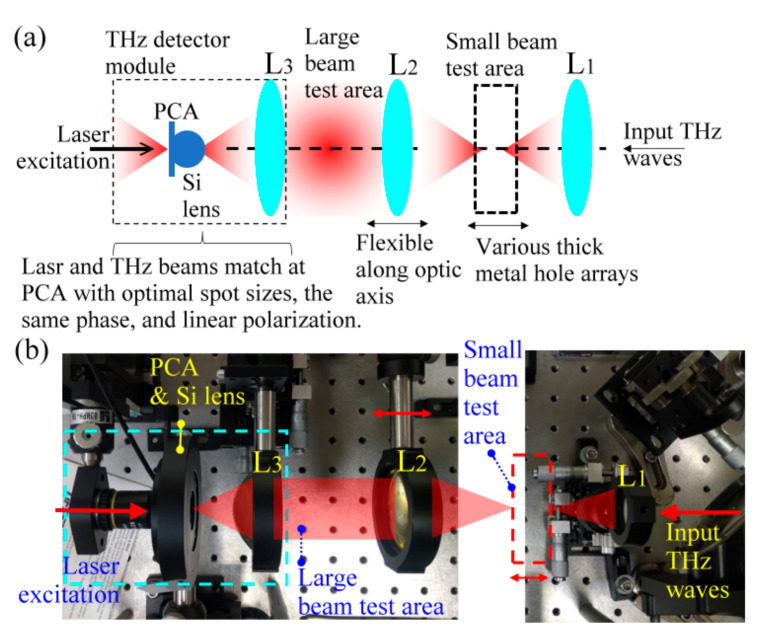
(**a**) Assemblies, configuration, and (**b**) photographs of THz wave detection in a polarization-selective THz-TDS system.

Re-emitting THz radiation at the output side of a thick MHA structure largely shifts the focused beam-waist position with a metal thickness such that it resembles the waveguide output end that is apart from the input coupling end. The optic parameters of THz wave detection for a thick MHA should be tunable along the optic axis to match various divergences of metal-hole re-emitting waves. Therefore, a waveguide-based THz time-domain spectroscopic (THz–TDS) system [20] was used to characterize the metal-hole-guided THz waves of MWW-HAs, MSAs, TH-, and OH-MHAs, which are listed in Table 1 and Table 2. The waveguide-based THz–TDS enables the optimized power detection for different metal thicknesses and beam sizes because the wave collimation assembly of the THz detector is flexible, as shown in Figure 4, which is opposite to the fixed THz–TDS optics of a traditional THz detector scheme [5]. THz lenses to guide and detect THz waves are expressed in Figure 4a and are denoted by L_1_, L_2_, and L_3_. Figure 4b shows photographs of their related configuration in the system. The L_1_ lens (EFL 25 mm) is used to focus the THz beam at the small beam test area. The L_2_ lens is flexible to allow it to move along the optic axis for the optimal power detection as sample structural thicknesses change. The large beam test area of the THz wave is located between L_2_ (EFL 50 mm) and L_3_ (EFL 50 mm). Those TH- and OH-MHAs with various thicknesses, 0.1–2 mm, can be characterized in power transmission, especially the OH-MHA thicknesses of 1 and 2 mm, which are larger than the Rayleigh range of 0.1–1 THz waves (i.e., 0.3–3-mm wavelengths). The THz emitter and detector are one pair of photoconductive antennas (PCAs) with the consistent *Z*-axial orientation of a dipole electrode. Thus, a linear polarization of THz radiation with a 20 dB distinction radiates along the dipole axis, which is defined as the *X* axis. The measured power spectra of MWW-HAs, MSAs, TH-, and OH-MHAs are thus polarization selective at the *X* axis. The detected THz wave power at the X axial polarization for different sample thicknesses can respond to the metal-hole rotating polarization angles, but not at the *X* axis. The THz detector module (Figure 4), constructed by the L_3_ (EFL 50 mm), one silicon lens, and one PCA, is excited by a laser beam and receives THz waves. To perform the optimal power detection, the laser and THz beams combine at the PCA with an optimal spot size, a consistent wave phase, and a consistent linear polarization.

## 3. Results

### 3.1. Spectral Features of Metal-Hole-Supported THz Resonance Waves

**Figure 5:**
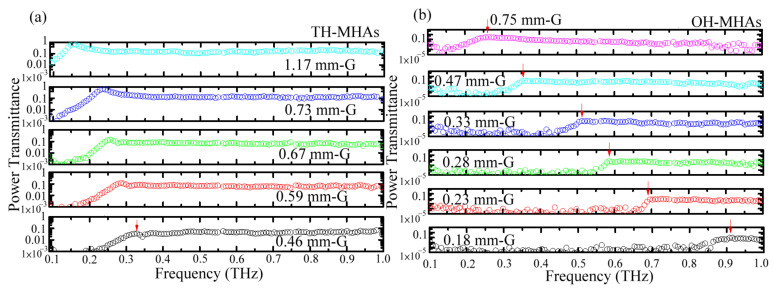
Power transmittance spectra for (**a**) TH- and (**b**) 1 mm thick OH-MHA structures.

Figure 5a,b shows the power transmittance spectra of TH- and OH-MHAs in 0.1–1 THz, respectively. Because the measured transmittance of metal-hole-rejected THz waves is much lower than that of the passed THz waves over two orders, logarithmic-scaled transmittance is thus used in Figure 5 instead of the linear-scaled one to fairly present wide-band transmittance observations. For each MHA, the THz wave frequency should be sufficiently high to pass through the MHAs, and this corresponds to a high-pass spectral feature. Thus, the high-frequency pass performance shows that the metal-hole guided THz waves have the TE mode feature, as expressed in Equation (6). Furthermore, the MWW-HA structure can also be considered as one THz plasmonic metamaterial due to the feature of a high-frequency pass spectrum, and the corresponding cut-off frequency represents a metal plasma frequency. The metal hole sizes can dilute the free electron density in the Drude model‘s dielectric function to artificially reduce a metal plasma frequency down to a THz frequency [1,8,10]. In Figure 5a, the wavelengths of spectral peaks for 0.59-, 0.67-, 0.73-, and 1.17-mm *G* TH-MHAs are 1.050, 1.177, 1.274, and 1.951 mm, respectively. The metal-hole widths, i.e., *G* values, and the measured half-wavelength magnitudes at the spectral peaks are similar. The spectral peaks of the TH-MHA structure can be expressed by TE resonance waves, as shown in Figure 2a and Equation (7), whose wavelengths are denoted as $\lambda_{c}$.

Although the transmittance of 0.46-mm *G* TH-MHA obviously reduces with low visibility of a spectral peak, the spectral-peak frequency near to the low-frequency rejection band, denoted by a red arrow in Figure 5a, can be considered as the resonance-wave frequency due to the approximate $\lambda_{c}$, based on Equation (7). For the OH-MHA structure with relatively low transmittance and *ρ_v_* values (Table 2), THz-wave frequencies in Figure 5b with the highest transmittance near to the low-frequency rejection bands can also be considered as the frequencies of metal-hole resonance waves. The metal-hole resonance waves of metal-slab-perforated TH- and OH-MHAs have the same spectral properties dependent on the *G* values, as illustrated in Figure 5. A blueshift of a metal-hole resonance wave occurs as the *G* value reduces, and the *G*-dependent variation trend of a blueshift is not hindered from the low transparency of THz resonance waves, which is resulted from large thicknesses, low porosity, or a small amount of metal holes. Metal-hole-supported THz resonance waves thus have loose criteria in geometry and contrast to the EOT of THz SRW observed in MHAs [4]. For example, metal hole sizes should be sufficiently small to allow metal holes perform the EOT of THz SRW that were found in the durations of waveform oscillation and surface evanescent field ranges [2]. 

Equation (2) and Figure 2 show that the metal-hole guided electric field amplitude composites the production of TE- and TM-mode amplitudes because of the symmetric metal structure of a circular hole. This means that linear-polarized THz waves along the *X* axis can pass through the metal holes (*Z* axis) synchronously possessing TE and TM modes in the *Y–Z* and *X–Z* planes, respectively, as shown in Figure 2. To derive the spectral features of TE and TM modes through a metal hole, the 0.25–0.78-mm metal-slit widths (i.e., the *G* values in Table 2) can be assumed as metal-hole diameters at the side view of an *X–Z* or *Y–Z* plane. The configuration of Figure 2 and the corresponding equations of the TE and TM modes in Equations (3)–(13) can be used to explain the metal-slit-guided TE and TM modes.

**Figure 6:**
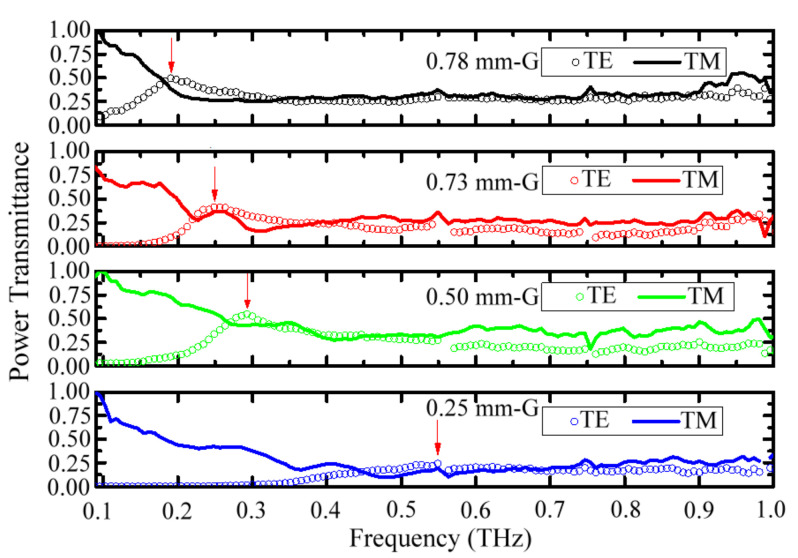
Power transmittance spectra for MSA structure.

Figure 6 illustrates the THz wave transmittance spectra for the TE and TM modes through the MSA structure. The TE modes of MSAs have a high-frequency pass feature, and the corresponding spectral peaks are near the low-frequency rejection bands, which are marked with red arrows, as shown in Figure 6. TM modes have high transmittances for THz wave frequency in the low-frequency rejection bands of TE modes. The transmission of MSA-TM waves is not constrained by the *G* values. This results from the *Z*-axial TM field, existing at the air–metal interfaces and independent from the *G* values, as shown in Equations (8)–(13) and Figure 2b. The transmittance product of the MSA-TE and TM modes in the spectra of Figure 6 can perform high-frequency pass spectra, consistent with those of the TH- and OH-MHA structures in Figure 5. This approves the rationality of Equation (2) to define amplitudes of the metal-hole-guided THz waves, indicating the modal mixing of TE and TM waves via the metal-hole guidance. The metal-hole transmittance spectra of the TH- and OH-MHA structures in Figure 5 are dominated by the corresponding TE waveguide modes.

**Figure 7:**
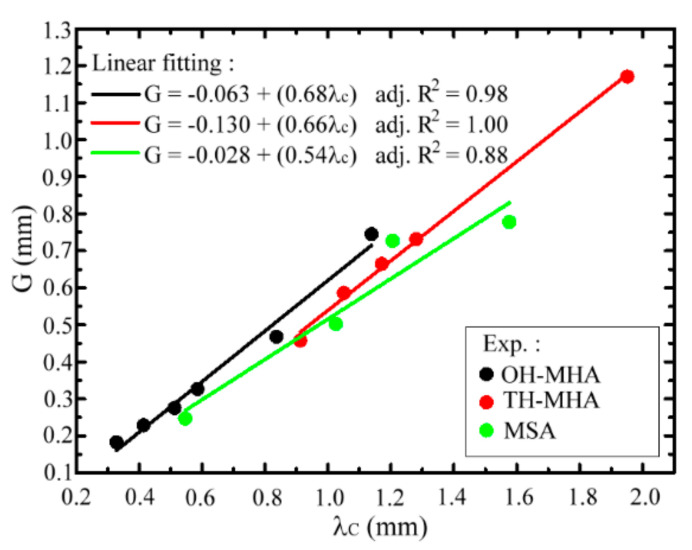
*G*-$\lambda_{c}$ relation for TH-, OH-MHA, and MSA structures.

Figure 7 summarizes the *G*-$\lambda_{c}$ relations of TH-, OH-MHAs, and MSAs, where $\lambda_{c}$ values are obtained from the measured spectral peaks of the high-pass spectra in Figure 5 and Figure 6. Proportional relations are observed between the *G* and $\lambda_{c}$ values, and the corresponding fitting lines with high adjusted coefficients are 0.88, 1.00, and 0.98 for MSA, TH-, and OH-MHA structures, respectively, as shown in Figure 7. The fitting-line slopes of the MSA, TH-, and OH-MHA structures are individually 0.54, 0.66, and 0.68, and the fitting-line slopes approximate the *G*/$\lambda_{c}$ ratios as ignoring the small values of *G* intercepts at fitting line equations. Although the spectral feature of high-pass spectra for TH- and OH-MHAs can be found from the TE mode spectra of MSAs, their numeric $\lambda_{c}$-*G* relations are clearly different in the *G*/$\lambda_{c}$ ratios.

Various *G*/$\lambda_{c}$ ratios between the MSA, TH-, and OH-MHA structures in Figure 7 respond to different approximation levels of the theoretical definition of a resonance wave in Equation (7), whose *G*/$\lambda_{c}$ ratio is 0.5. TH-, and OH-MHA structures have lower approximation percentages of 75% and 73%, respectively, compared with the 92% approximation percentage of the MSA structure. The discrepancy of a *G*/$\lambda_{c}$ ratio comes from the different precisions of measured $\lambda_{c}$ values or spectral peak frequencies in Figure 5 and Figure 6. 

In the experiment, the *X*-axial linear polarization input TH- and OH-MHAs certainly rotates with a *Z-*axial azimuth angle when the THz resonance waves are guided and supported through metal holes, as shown in Figure 2 [21]. The transmittance of a rotated resonance wave evidently reduces because its polarization azimuth deviates from the dipole electrode in a THz–TDS system, i.e., the *X* axis in the experiments. Consequently, the measured $\lambda_{c}$ value of a spectral peak changes and does not exactly match a metal-hole width, *G*, with a 0.5-*G*/$\lambda_{c}$ ratio. Furthermore, for one *G* value of 0.45–0.75 mm, observed in Figure 7 as an example, the corresponding $\lambda_{c}$ values of OH-MHAs are slightly smaller than those of TH-MHAs, but those of TH-MHAs and MSAs are similar. Table 2 shows that the metal thickness, *T*, of an OH-MHA structure is larger than those of TH-MHA and MSA structures. The metal-hole thickness critically influences the measured spectral peak frequency; in particular, a large thickness can slightly shift the spectral peak toward a high-frequency range, which is not found from the metal thickness-dependent spectral properties of THz-SRW EOT [22].

**Figure 8:**
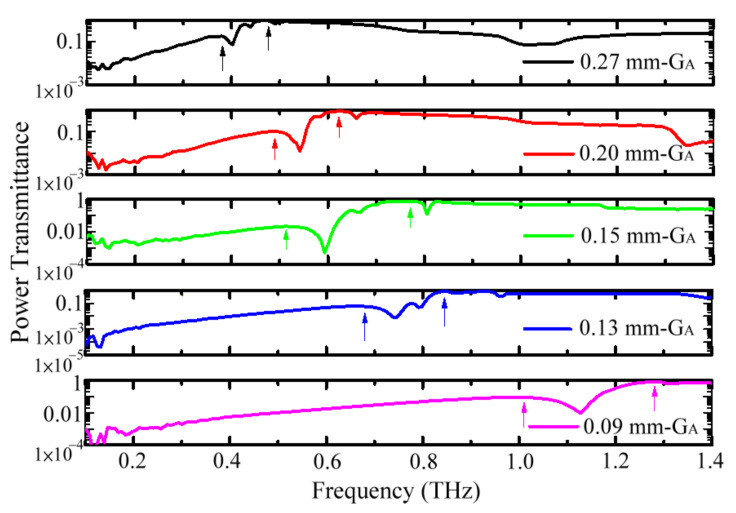
Power transmittance spectra of MWW-HA structure.

Figure 8 illustrates that the high-pass spectral feature of metal-hole-guided THz waves can also be performed by MWW-HAs, as shown in Figure 1 and Table 1, but spectral peaks split, as denoted by the two arrows. The first and second spectral peaks are located at low- and high-frequency ranges, respectively, possessing low and high transmittances. The peak splitting on metal-slab-perforated MHAs, such as TH-MHAs, is also demonstrated with certain oblique angles of input THz waves [16]. However, for measuring MWW-HAs, THz waves are input on the *X–Y* plane of an MWW-HA with a 90° incident angle, i.e., along the *Z* axis in Figure 1. The mechanism of spectral peak splitting between TH-MHA and MWW-HA is different due to their different unit-cell structures, as shown in Figure 1 and Figure 3a.

The metal holes of an MWW-HA unit cell are constructed by woven metal wires with straight and bent sections, as observed in the *X–Z* and *Y–Z* planes and shown in Figure 1. By contrast, the metal holes of metal-slab-perforated MHAs are planar for TH- and OH-MHA structures, as shown in Figure 3a,b, respectively. Based on the principle of metal-hole-guided resonance waves, the perforated metal-hole widths, i.e., the *G* values, determine the spectral peak frequencies, as shown in Figure 7. The blueshift trend with the decreased metal-hole widths of TH- and OH-MHAs can also be found from the MWW-HAs in Figure 8. The metal-hole widths of MWW-HAs are the *G_A_* values, as shown in Figure 1.

**Figure 9:**
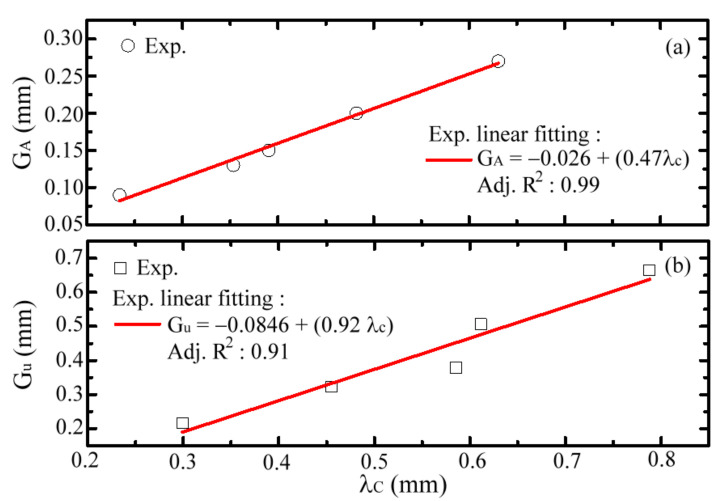
(**a**) *G_A_*-$\lambda_{c}$ and (**b**) *G_u_*-$\lambda_{c}$ relations of MWW-HA structure.

The second spectral peaks of MWW-HAs have the highest transmittance and are summarized to correlate the *G_A_* values, as shown in Figure 9a. The corresponding peak wavelength, $\lambda_{c}$, is proportional to the *G_A_* value, where the fitting line has a high adjusted coefficient of determination, as shown by the red line in Figure 9a, i.e., adj. R^2^ = 0.99. The *G_A_*/$\lambda_{c}$ ratio or measured slope of the fitting line in Figure 9a is 0.47. Compared with the 0.5-*G*/$\lambda_{c}$ ratio in Equation (7), the approximation percentage of an MWW-HA is up to 94%, much higher than those of TH- and OH-MHA structures, as shown in Figure 7. The metal-hole-rotated polarization angle through the MWW-HA structure is certainly smaller than those of TH- and OH-MHA structures. This means that the polarization performance of MWW-HA approaches that of the TE resonance wave of MSA and the theoretical TE mode of a metal-hole waveguide mode, as shown in Equations (3)–(7) and Figure 2a.

Figure 9b summarizes the resonance wavelengths ($\lambda_{c}$) of the first spectral peaks in Figure 8 and correlates $\lambda_{c}$ values with *G_u_* values. A proportional relation exists between $\lambda_{c}$ and *G_u_*, whose fitting line has the high adjusted coefficient of determination, as shown by the red line in Figure 9b, i.e., adj. R^2^ = 0.91. The *G_u_*/$\lambda_{c}$ ratio or measured slope of the fitting line in Figure 9b is 0.92. The $\lambda_{c}$ value at the first spectral peak is similar to the *G_u_* value, with approximately 8% deviation. This means that THz wave resonance possibly occurs to cover one unit-cell hole of *G_u_* to propagate through the MWW-HA structure. Thus, the THz waves at the first spectral peaks can be explained by the TE mode resonance of planar MHAs, as shown in Equations (3)–(7), when the boundary range of a metal hole is double. The first and second spectral peaks of MWW-HAs belong to metal-hole resonance waves within the ranges of *G_A_* and *G_u_*, respectively.

### 3.2. Transmittance Performance of Metal-Hole Resonance Waves

**Figure 10:**
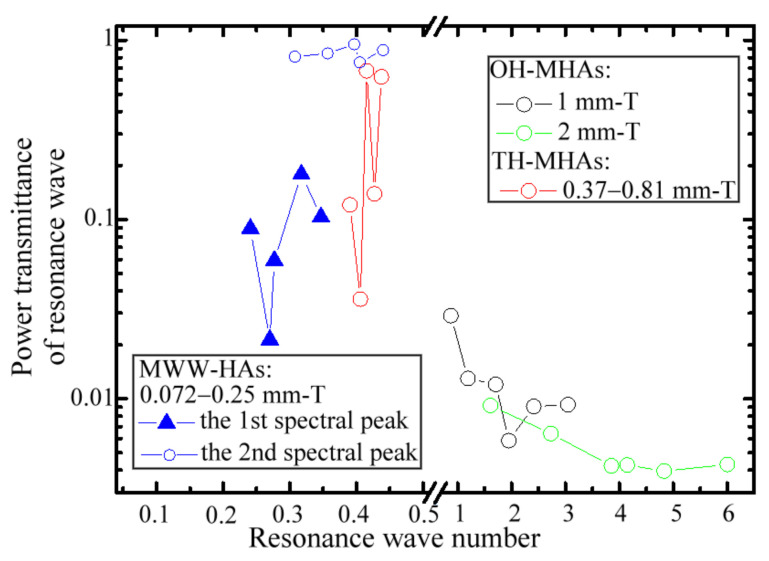
Relation of power transmittances and metal-hole waveguide lengths for resonance wave transmission through TH-, OH-MHA, and MWW-HA structures.

To account for the propagation lengths of different resonance waves, i.e., various $\lambda_{c}$ values, within one metal thickness exactly, the wave number of a resonance wave longitudinally inside the metal, equaling *T*/*λ_c_*, is considered for a waveguide length of a metal-hole structure. Figure 10 summarizes the relation between the transmittances and wave numbers of resonance waves, passing through MWW-HA, TH-, and OH-MHA structures. The resonance waves are specified from the spectral peaks of Figure 5 and Figure 8. Based on a polarization-selective THz-TDS system (*X* axis), the 1- and 2-mm thick OH-MHAs shown in Figure 3b are designed to observe the transmittance variation for the metal-hole waveguide length above one resonance wave number. The resonance-wave transmittance of 1-mm thick OH-MHAs, denoted by black circles in Figure 10, sharply reduces from 0.029 to 0.006 but is sustained at 0.009 for the waveguide lengths, respectively, at 1–2 and 2–3 wave numbers. For the 2-mm thick OH-MHAs, the resonance-wave transmittance denoted by green circles in Figure 10 gently decay from 0.009 to 0.004 for a waveguide length variation of 1–6 wave numbers.

Although transmittance fluctuation occurs in a polarization-selective THz-TDS system for the variation of a metal-hole waveguide length less than one wave number, transmittance decay becomes evident for the variation of a metal-hole waveguide length of more than one wave number, as shown in Figure 10. Thus, the transmittance decay of metal-hole waveguiding modes follows the character of a bulk waveguide. Based on a polarization-selective THz-TDS system (*X* axis), MWW-HAs and TH-MHAs were used in the experiment to observe the transmittance difference of THz resonance waves less than one wave number of a metal-hole waveguide length. For the range of a metal-hole waveguide length in 0.39–0.43 wave numbers, the resonance transmittance of TH-MHAs (the red-circle data) seriously fluctuates in the range of 0.04–0.67. However, in the same metal-hole waveguide length of 0.39–0.43 wave numbers, the transmittance fluctuation range of MWW-HA resonance waves at the second spectral peak is only 0.75–0.94, which is contrarily small and denoted by the blue-circle data in Figure 10. The MWW-HA structure performs not only the high transmittance but also the stable polarization transmission of the resonance waves by adjusting the *G* and *T* values.

Based on a polarization-selective THz-TDS system (*X* axis), the small transmittance fluctuation with a metal-hole waveguide length means that the azimuth of linear polarization though an MWW-HA structure slightly changes, leading to a small rotation angle of the output polarization. Comparing their unit-cell configurations in Figure 1 and Figure 3a reveals that the round metal edge of an MWW-HA structure is one important factor to make the effective optical length of a metal-hole waveguide much smaller than the physical metal thickness. By contrast, the right-angle metal edge of an TH-MHA structure has an effective optical length of resonance-wave guidance approximately equal to a physical metal thickness. For one resonance wave passing through one metal structural thickness, the effective metal-hole thickness of a right-angle metal edge is larger than that of a round metal edge. Eventually, the TH-MHA structure causes the polarization azimuth of the resonance wave to be sensitive to the variation of a metal thickness. However, the resonance wave transmittance randomly varies with *T*, *G*, and *λ_C_* values due to rotated polarization, which is consistent with the thickness-dependent spectral peaks of THz-SRW EOT [16]. Based on a polarization-selective THz-TDS system (*X* axis), the polarization-rotation-inducing transmittance fluctuation of resonance waves is also found along the 1- and 2-mm thick OH-MHAs at the waveguide-length segments of 1–2, and 2–3 wave numbers, as shown in Figure 10.

Furthermore, MWW-HAs have very high porosities (*ρ_v_*) due to the four metal-hole amounts within the unit-cell structure. Table 1 shows that the *ρ_v_* range of MWW-HA is 71–77%. By contrast, Table 2 shows that the *ρ_v_* range of TH-MHA is 10.3–17.4%. MWW-HAs can perform the highest transmittance, which cannot be achieved by TH-MHAs despite an approximate metal-hole waveguide length. As expressed by the blue-triangle data in Figure 10, the resonance waves of MWW-HA at the first spectral peaks have a considerably low transmittance of 0.02–0.18, which is much lower than that of the second spectral peaks, i.e., the blue-circle data in Figure 10. The *X–Y* boundary range of the *G_u_* value in Figure 1a,b shows that one metal-wire cross section interacts with resonance waves to result in a high propagation loss.

## 4. Discussion

### 4.1. Spectral-Peak Splitting of a Resonance Wave

**Figure 11:**
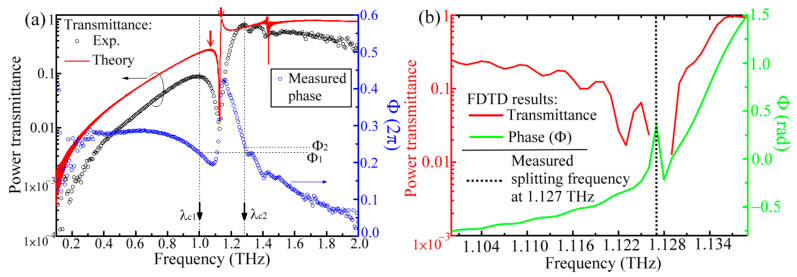
(**a**) Power-transmittance and phase spectra for the 0.09-mm *G_A_* MWW-HA. (**b**) FDTD calculation results of power transmittance and phase in 1.1–1.139 THz for the 0.09-mm *G_A_* MWW-HA.

To explain the spectral peak splitting of an MWW-HA structure, the waveguide electric field through one structural unit (Figure 1) was calculated by the 3D finite-difference time-domain (FDTD) method with a periodic-structural boundary in the *X*–*Y* plane. The MWW-HA with a 0.09-mm *G_A_* value (Table 1) is taken as an example in the discussion. The black circles in Figure 11a illustrate the measured power transmittance spectrum replotted from the magenta curve in Figure 8. In the experiment, the spectral peak of the 0.09-mm *G_A_* MWW-HA splits at 1.127 THz with one each of low- and high-transmittance spectral peaks, which are respectively located at the rejection and pass bands. The splitting frequency at 1.127 THz corresponds to the spectral dip, measured at the transmittance spectrum curve of black circles in Figure 11a. The FDTD spectral calculation can also identify the peak splitting effect according to the modeling parameters in Table 1. To ascertain the peak splitting effect at the 1.127 THz frequency in the FDTD calculation, the *G_A_* value should be enlarged from 0.09 mm to 0.111 mm. The other MWW-HA parameters of 0.036-mm *M_w_*, 0.07-mm *R_c_*, and 36.22° *θ* were not adjusted (Table 1). As the metal holes of the *G_A_* apertures were constructed by two pairs of cross woven metal wires without being exactly fixed, mechanical deformation is possible for such a 0.021-mm variation of the *G_A_* value.

The FDTD-calculated transmittance spectrum is depicted by the red curve in Figure 11a. The calculated first and second spectral peaks, denoted by the two red arrows, occur, respectively, at 1.069 and 1.141 THz in Figure 11a. However, the measured first and second spectral peaks are at 1.002 and 1.283 THz, individually. The calculated spectral-peak frequencies deviate from the measured ones, respectively, with 67 and 142 GHz. The calculated split peaks are closer to the 1.127 THz splitting frequency than to the measured ones. The Fourier transformation principle indicates that the spectral sharpness at the splitting frequency is critically dominated from the uniformity of the periodically woven metal wires within a THz wave spot. Despite the slight inaccuracy on the resonance wave frequencies at the first and second spectral peaks given the structural variation among the unit cells, high visibility with apparent transmittance discrepancy between the two peaks is found from the FDTD calculation and measured results.

As the aperture *G_A_* values are tuned in the FDTD calculation, the spectral shift effect for the two spectral peaks of one MWW-HA structure can be ascertained, and the outcomes are consistent with the trend of the measured results in Figure 8. The FDTD calculation results also show that the wavelengths of the resonance waves, $\lambda_{c}$, at the first and second spectral peaks are individually proportional to the *G_u_* and *G_A_* values, thereby approaching the linear fitting lines in Figure 9. That is, the *G_u_/*$\lambda_{c}$ and *G_A_/*$\lambda_{c}$ ratios calculated by the FDTD method also approach 0.5 and 1.0, respectively, as outcomes established when ignoring the small *G_u_* and *G_A_* intercepts of linear fitting. Therefore, the FDTD calculation results prove the rationality of the waveguide electric field, as expressed in Equations (1)–(13).

### 4.2. Interaction between Woven Metal Wires and a Resonance Wave

To specify the interaction between the woven metal wires and a resonance wave, phase retardations of THz field oscillation at the first and second spectral peaks were monitored from the measured time-domain waveforms and denoted as *Ф*_1_ and *Ф*_2_, respectively. For the example of a 0.09-mm *G_A_* MWW-HA, the phase retardation spectrum in 0.1–2 THz is measured and expressed by the blue circles in Figure 11a. The phase retardations of the two resonance waves, *Ф*_1_ and *Ф*_2_, are measured at 1.002 and 1.283 THz. Figure 11b further presents the FDTD calculated transmittance and phase spectra of a 0.09-mm *G_A_* MWW-HA in the narrow bandwidth of 1.1–1.139 THz to observe transmittance performance around the peak-splitting frequency at 1.127 THz, i.e., the dotted line in Figure 11b. The corresponding transmittance and phase spectra are illustrated, respectively, by the red and green curves in Figure 11b. However, the transmittance of 1.126–1.129 THz waves cannot be resolved from the FDTD method due to the extremely weak transmission, where the frequency range covers the measured splitting frequency of 1.127 THz. In addition, a spectral phase peak at 1.126–1.129 THz exactly indicates the phase spectral feature of a resonant medium with strong absorption loss [23]. This proves that the THz wave at a splitting frequency of 1.127 THz cannot be supported within the metal holes of an MWW-HA. Therefore, the structural model presented in the literature [18], including the geometric factors, measurement, and analysis methods, also cannot express the fundamentals of a THz resonance field locally guided by the metal holes of an MWW-HA.

**Figure 12:**
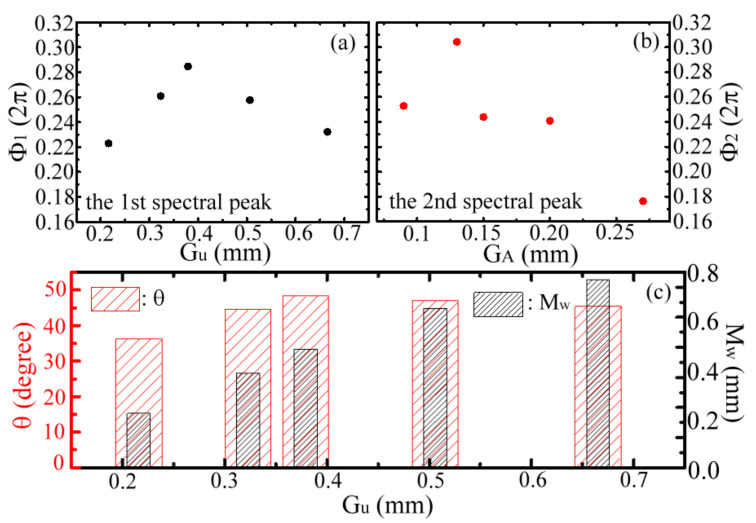
(**a**) *G_u_*- and (**b**) *G_A_*-dependent phase retardations of different MWW-Has, respectively, at their first and second spectral-peak frequencies. (**c**) *G_u_*-dependent *Mw* and *θ* for the MWW-HA structure.

Figure 12a,b summarizes the measured *Ф*_1_ and *Ф*_2_ values relating to the *G_u_* and *G_A_* values, respectively, for all the MWW-HAs in Table 1. The highest *Ф*_1_ value is achieved by the 0.15-mm *G_A_* MWW-HA, with a 0.379-mm *G_u_* value (Figure 12a), but for the highest *Ф*_2_ value, the *G_A_* value should shrink to 0.13 mm (Figure 12b). Thus, the resonance fields of the first and second spectral peaks are intrinsically different. For example, the 0.09-mm *G_A_* MWW-HA has the lowest *Ф*_1_ value according to a 0.216-mm *G_u_* value among the MWW-HAs (Figure 12a), but its *Ф*_2_ value is contrarily high at the second place (Figure 12b). High phase retardation with a high *Ф*_1_ or *Ф*_2_ value represents a strong interaction between woven metal wires and metal-hole resonance waves. By contrast, the weak interaction of woven metal wires is characterized by low *Ф*_1_ or *Ф*_2_ values.

As the THz resonance wave at the first spectral peak transversely interacts with one woven metal wire between two adjacent *G_A_* apertures (Figure 9b), the cross-section factors, *M_w_* and *θ*, of a woven metal wire can determine the order of the phase retardation (*Ф*_1_). According to the MWW-HA information in Table 1, Figure 12c shows that the values of the *M_w_* and *θ* parameters are related to the *G_u_* values. Relative to the *G_u_*-dependent variation trends in Figure 12a,c, the parameter of the bending angle, *θ*, can determine the order of the *Ф*_1_ value instead of the wire diameter, *M_w_*. To model the metal holes of an MWW-HA structure, the *θ* parameter of a woven metal wire, as shown in Table 1, should be correlated to the parameters of *R_C_*, *M_w_*, and *G_u_* on the basis of Equation (14),

(14) $$\theta =2\left[\sin^{-1}\frac{R_{C}-0.5M_{w}}{\sqrt{{\left(R_{C}-M_{w}\right)}^{2}+{\left(\frac{\Lambda }{4}\right)}^{2}}}-\sin^{-1}\frac{R_{C}-M_{w}}{\sqrt{{\left(R_{C}-M_{w}\right)}^{2}+{\left(\frac{\Lambda }{4}\right)}^{2}}}\right],$$

where Λ = *G_u_ + M_w_*. For each set of *M_w_* and *G_u_* (or *G_A_*), the *θ* parameter can optimize the tangential combination among metal wires to weave four rectangular holes as one unit cell (Figure 1b,c). 

The factors *M_w_* and *θ* also affect the phase retardation of the metal-hole-guided resonance wave at the second spectral peak (*Ф*_2_), but this outcome is only found on two MWW-HAs whose *G_A_* values are 0.09 and 0.13 mm (Figure 12b,c). The *G_A_* values larger than 0.13 mm, i.e., 0.15–0.27-mm *G_A_*, critically dominate the *Ф*_2_ value with an inverse proportionality. As presented in Figure 12b, the *Ф*_2_ value obviously decreases with the increase of the *G_A_* value from 0.13 mm to 0.27 mm.

### 4.3. Power Flow Distributions of Resonance Waves

**Figure 13:**
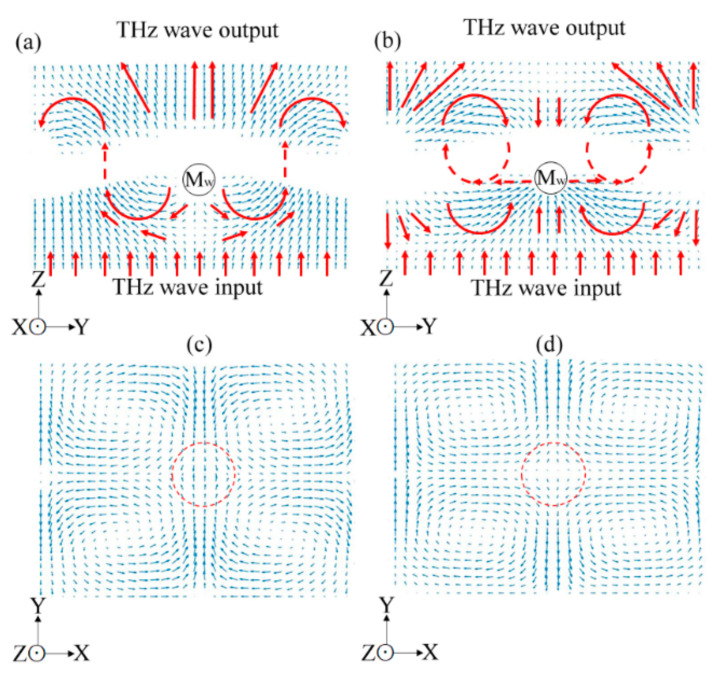
Power flow of the 0.09-mm *G_A_* MWW-HA. Side views at (**a**) 1.069 and (**b**) 1.141 THz. Top views at (**c**) 1.069 and (**d**) 1.141 THz.

The power flows or Poynting vectors of the 0.09-mm *G_A_* MWW-HA are calculated by the FDTD method. Figure 13a,b, respectively, show the side views of the power-flow distributions at 1.069 and 1.141 THz, which are the metal-hole resonance waves at the first and second spectral peaks and denoted by the red arrows in Figure 11a. The side-view plane locates at the unit-cell center of the *X* axis, and one metal-wire cross section locates at the middle of the *Y* axis (Figure 1b), which is called the middle-woven-metal wire and denoted by the *M_w_* diameter in Figure 13a,b. The red arrows in Figure 13a,b with solid red lines are emphasized for the calculated results of the Poynting vectors, which, in turn, are presented as blue arrows. Red arrows with dashed lines indicate the possible paths of power flow alongside the metal wires given the absence of a Poynting vector inside the metal wires.

Figure 13a shows the obvious power scattering around the middle-woven-metal wire for the 1.069 THz resonance wave. The partial scattering power of the 1.069 THz wave consequently passes through the two *G_A_* apertures to arrive at the output end face. By contrast, the input power flow of the 1.141 THz resonance wave concentrates at the middle-woven-metal wire to circulate through the *G_A_* apertures, as illustrated in Figure 13b. Such a power scattering effect strongly decays the output power of the 1.069 THz resonance wave. Conversely, the propagation of the 1.141 THz resonance wave is fluent without obvious power-scattering loss. The side-view power flow explains why the transmittance of the first spectral peaks is much lower than that of the second spectral peaks, as shown in Figure 10.

Figure 13c,d presents the top views of the power flow at 1.069 and 1.141 THz frequencies, respectively. The top-view plane is located in the output-end range and at *Z* = 2*M_w_* (Figure 1c). For the 1.069 THz resonance wave, the power flow cross linking four *G_A_* apertures can be found from the Poynting vectors within the red circle in Figure 13c. At the same red-circle location, the Poynting-vector magnitudes of the 1.141 THz resonance wave in Figure 13d are much smaller than those of the 1.069 THz resonance wave. That is, the power flow of the 1.141 THz wave mainly circulates within each *G_A_* aperture and without any apparent cross-linking effect. The power flow of the top view indicates that the resonance waves at the first spectral peaks have stronger interaction with the woven metal wires of MWW-HA than that of the second spectral peaks. Therefore, the measured *Ф*_1_ value is approximately proportional to the bending angle, *θ*, of a woven metal wire that is complexly dependent on the *M_w_* and *R_C_* parameters of a woven metal wire and a metal-hole width of *G_u_* (Equation (14)).

Furthermore, the top-view Poynting vectors of the two metal-hole-guided resonance waves through an MWW-HA clearly shows that polarization rotating on the *X*–*Y* plane results from the power circulation effect due to metal-surface-reflected guidance. The power circulation is expressed by the blue arrows of the Poynting vectors in Figure 13c,d. As the Poynting vector, i.e., the propagation direction, rotates with the *Z* axis, the electric field direction also rotates. However, the power circulation effect of MWW-HA is weak at the metal-hole centers; contrarily, the strongest power circulation effect occurs at the metal wire surface. Figure 13c,d therefore demonstrates that the rotated polarization of metal-hole-guided resonance waves really exists inside the metal holes of MWW-HA, but the rotating magnitude is much smaller than that of OH- and TH-MHAs, which are observed by the polarization-selective THz-TDS system in Figure 10. 

As the woven metal wires constitute the four rectangular holes of the MWW-HA unit cell, the unit-cell-hole width, *G_u_*,should be additionally specified (Table 1) and varies from the defined *G* values of the planar TH- and OH-MHAs (Table 2). The wire bending configuration of the middle-woven metal wire is inverse to that of the adjacent ones, while reviewing at the *X*–*Z* planes and the 3D version (Figure 1b,c). Therefore, the THz waves that are rejected from the *G_A_* apertures can transversely extend, cross the adjacent apertures, and consequently cover the *G_u_* unit-cell holes. Such transverse extensions of the *G_A_* aperture-rejected waves eventually achieve resonance transmission according to Equations (3)–(7) and a *G_u_* boundary range. However, the corresponding transmittance of the *G_u_* resonance waves is still much lower than that of the *G_A_* resonance waves, given the resistance of a middle-woven-metal wire.

### 4.4. Characterization and Comparison

Metal-hole supported THz resonance waves with transmission spectral peaks mainly result from four types of MHA structure, including MWW-HAs, Metal-slab-perforated HAs [8,24,25,26,27], MHA patterns on semiconductor slabs [5,28,29], and polymer membranes [30,31,32]. They are compared in Table 3 for the characterizations of high THz transparency > 90%, linear polarization maintenance, free dielectric substrate loss, the EOT of THz SRW, and bendable and deformable features. Both MWW-Has and metal-slab-perforated HAs are freestanding constructions without dielectric substrate loss, which is resulted from interface reflection and intrinsic dielectric absorption [33]. For the sufficiently large metal holes [2,8,24] or oblique THz wave input [16], metal-slab-perforated HAs can perform EOT of THz SRW at transmission spectral peaks; however, the highest transmittance above 90% is not yet reported in the literatures. For the same metal thickness, THz plasmonic metamaterials based on MWW-HAs are more bendable than those based on metal-slab-perforated HAs (i.e., OH- and TH-MHAs) because of the high porosity of MWW-HA (Table 1 and Table 2). Based on the specifically thin metals, the EOT of THz SRW can be found from the transmission spectral peaks of MHA patterns on semiconductors and polymer membranes. High THz transparency > 90% of MHA patterns on semiconductors has been demonstrated, but the metal holes were asymmetric for the structural criterion [5,28]. This thus proves that low peak transmittance for the symmetric metal holes of MHA patterns on semiconductors probably results from the polarization rotation at a polarization-selective THz-TDS system. However, the polarization performance of MHA patterns on polymer membranes has not yet been discussed. The presented polymer membranes of MHA substrates have extremely small dielectric thicknesses and lower THz dispersion (or refractive indices) of THz waves [30,31,32], compared to the semiconductor substrates of MHAs. The corresponding dielectric absorption and interface-reflection losses on polymer membrane-based MHAs are certainly low. Polymer membrane-based THz plasmonic metamaterials are bendable and deformable, much like MWW-HAs. However, the electroforming metal patterns of MHAs easily fall off the polymer membranes due to weak attachment while bending or deforming the MHA polymer membranes. MWW-HAs are therefore the more robust THz plasmonic metamaterials to deform for various constructions and are critically fundamental for achieving novel applications of THz metamaterials. For pipe- and cone-shaped MWW-Has, as examples, the anti-resonance or resonance waves at the first spectral peak can be reflected from MWW-HAs to propagate along the hollow core of pipe- and cone-shaped MWW-HAs. The high porosity of MWW-HA eventually leads gaseous analytes to interact with MWW-HA-guided THz waves with a sufficiently long distance for gas-sensing purposes, which cannot be achieved by the available THz metamaterial fibers based on a polymer substrate [15].

## 5. Conclusions

THz waves guided through and supported by the metal holes of an MWW-HA structure are experimentally investigated in the transmittance spectra and compared with the standard structures of MSA and MHA. The metal-hole-guided THz fields with high-frequency pass spectra represent the electric field mixing between the TE and TM waveguide modes because of a symmetric metal boundary. The measured spectral-peak frequencies near the low-frequency rejection bands can be specified as resonance waves inside metal holes because the very low magnitudes of propagation constants approximate zero. Metal-hole resonance waves are fundamentally dominated by TE waveguide modes because a metal-hole width, *G*, is proportional to the wavelength of a resonance wave, *λ_c_*, with a *G*/*λ_c_* ratio of 0.5. Relative to the TH- and OH-MHAs, the MWW-HA structure has the highest approximation percentage to the 0.5-*G*/*λ_c_* ratio because the output polarization of the MWW-HA-guided resonance waves have small rotation angles, approaching a polarization-maintained performance. When the metal-hole resonance waves in 0.1–1.5 THz pass through an MWW-HA structure, spectral-peak splitting occurs because of metal holes constructed by woven-metal wires instead of perforating metal slabs. The metal holes constructed by woven metal wires achieve high unit cell porosity, thereby leading to the highest transmittance of up to 0.94 in the experiments. The round metal edges of the woven metal wires can miniaturize the azimuth variation of the input linear polarization given the small effective optical length on the metal surface. Given the high porosities and round-metal edges, the MWW-HA structure has low resistance to guide and support THz resonance waves, consequently maintaining the input power and polarization. However, such a low resistance of the MWW-HA-resonance-wave propagation only performs at the second spectral peak. The power of the MWW-HA resonance wave at the first spectral peak is very weak at the metal-hole rejection band, whose transmittance is one order lower than that of the second spectral peak. For the first-peak resonance wave, the calculated Poynting vectors show strong interaction at the crossed middle-woven-metal wires of an MWW-HA structure. Unlike metal-slab-perforated metal holes (such as the TH- and OH-MHA structures), the MWW-HA structure involves middle-woven-metal wires to weave the unit cell. The bending angle, *θ*, of a woven metal wire is found to be the critical factor, and the *θ* angle value is approximately proportional to the interaction strength of the first-peak resonance waves as measured by the phase retardation. The resonance waves at the second peaks realize the MWW-HA structure as one qualified medium of metal-hole waveguide modes with considerably higher THz transparency and pure linear polarization. 

## Figures and Tables

**Table 1:** Metal-wire-woven hole arrays (MWW-HAs).

*G_A_* (mm)	*M_w_* (mm)	*G_u_* (mm)	*R_C_* (mm)	*θ* (°)	*ρ_v_* (%)
0.27	0.125	0.665	0.233	45.43	74
0.20	0.106	0.506	0.213	47.04	71
0.15	0.079	0.379	0.160	48.29	71
0.13	0.063	0.323	0.124	44.61	73
0.09	0.036	0.216	0.070	36.22	77

**Table 2:** Metal-slab-perforated hole arrays.

Unit Cell	*G* (mm)	*M_Y_* (mm)	*M_X_* (mm)	*T* (mm)	*ρ_v_* (%)	Section
Two-hole unit	1.17	1.51	0.89	0.81	17.4	- -
	0.73	1.01	0.57	0.56	16.3	
	0.67	1.05	0.61	0.55	13.3	
	0.59	0.94	0.54	0.41	13.1	
	0.46	0.86	0.50	0.37	10.3	
One-hole unit	0.75	1.03		1.00 and 2.00	5.5	(1)
	0.47	1.17			4.9	(2)
	0.33	0.84			5.4	(3)
	0.28	0.76			4.0	(4)
	0.23	0.69			5.0	(5)
	0.18	0.61			5.0	(6)
One-slit unit	0.78	0.73 0.56 0.51 0.55	- -	0.10	52.0	- -
	0.73			0.39	56.0	
	0.50			0.20	50.0	
	0.25			0.69	31.0	

**Table 3:** Characterizations of metal-hole supported THz resonance waves at transmission spectral peaks. (✓: working, ✗: not working).

Metal Hole Array (MHA)	High THz Transparency > 90%	Linear Polarization Maintenance	Free Dielectric Substrate Loss	EOT of THz SRW	Bendable and Deformable Features
MWW-HAs	✓	✓	✓	✗	High, robust metal structures
Metal-slab-perforated HAs [8,24,25,26,27]	✗	✗	✓	✓	Low
MHA patterns on semiconductor slabs	✓ (Asymmetric holes) [5,28]	✗	✗ (High loss)	✓	✗
	✗ (Symmetric holes) [29]				
MHA pattern on polymer membranes [30,31,32]	✓	- - Not available	✗ (Low loss)	✓	High, weak metal structures

## Data Availability

Not applicable.
